# Imazapic Herbigation for Egyptian Broomrape (*Phelipanche aegyptiaca*) Control in Processing Tomatoes—Laboratory and Greenhouse Studies

**DOI:** 10.3390/plants10061182

**Published:** 2021-06-10

**Authors:** Yaakov Goldwasser, Onn Rabinovitz, Zev Gerstl, Ahmed Nasser, Amit Paporisch, Hadar Kuzikaro, Moshe Sibony, Baruch Rubin

**Affiliations:** 1 R.H. Smith Institute of Plant Science & Genetics in Agriculture, R.H. Smith Faculty of Agriculture, Food and Environment, The Hebrew University of Jerusalem, P.O. Box 12, Rehovot 76100, Israel; kapitush@gmail.com (A.P.); hadar_koz@walla.co.il (H.K.); moshe.sibony@mail.huji.ac.il (M.S.); rubin@mail.huji.ac.il (B.R.); 2 The Agricultural Extension Service, The Ministry of Agriculture, P.O. Box 28, Bet Dagan 50200, Israel; onnrab@gmail.com; 3 Institute of Soil, Water and Environmental Sciences, Volcani Center, ARO, P.O. Box 6, Bet Dagan 50250, Israel; zgerstl@volcani.agri.gov.il (Z.G.); nasser@volcani.agri.gov.il (A.N.)

**Keywords:** chemigation, drip irrigation, Egyptian broomrape, herbicide, imazapic, parasitic plants, tomato, weed control

## Abstract

Parasitic plants belonging to the Orobanchaceae family include species that cause heavy damage to crops in Mediterranean climate regions. *Phelipanche aegyptiaca* is the most common of the Orobanchaceae species in Israel inflicting heavy damage to a wide range of broadleaf crops, including processing tomatoes. *P. aegyptiaca* is extremely difficult to control due to its minute and vast number of seeds and its underground association with host plant roots. The highly efficient attachment of the parasite haustoria into the host phloem and xylem enables the diversion of water, assimilates and minerals from the host into the parasite. Drip irrigation is the most common method of irrigation in processing tomatoes in Israel, but the delivery of herbicides via drip irrigation systems (herbigation) has not been thoroughly studied. The aim of these studies was to test, under laboratory and greenhouse conditions, the factors involved in the behavior of soil-herbigated imazapic, and the consequential influence of imazapic on *P. aegyptiaca* and tomato plants. Dose-response Petri dish studies showed that imazapic does not impede *P. aegyptiaca* seed germination and non-attached seedlings, even at the high rate of 5000 ppb. Imazapic applied to tomato roots inoculated with *P. aegyptiaca* seeds in a PE bag system revealed that the parasite is killed only after its attachment to the tomato roots, at concentrations as low as 2.5 ppb. Imazapic sorption curves and calculated Kd and Koc values indicated that the herbicide Kd is similar in all soils excluding a two-fold higher coefficient in the Gadash farm soil, while the Koc was similar in all soils except the Eden farm soil, in which it was more than twofold lower. In greenhouse studies, control of *P. aegyptiaca* was achieved at >2.5 ppb imazapic, but adequate control requires repeated applications due to the 7-day half-life (t_1/2_) of the herbicide in the soil. Tracking of imazapic in soil and tomato roots revealed that the herbicide accumulates in the tomato host plant roots, but its movement to newly formed roots is limited. The data obtained in the laboratory and greenhouse studies provide invaluable knowledge for devising field imazapic application strategies via drip irrigation systems for efficient and selective broomrape control.

## 1. Introduction

Parasitic plants account for approximately one percent of angiosperm species and are present in 275 genera belonging to 28 botanical families [1]. Some of the parasitic plants are important agricultural weeds, infest a varied range of crops worldwide, and pose a threat to the food security of many communities [2,3,4]. The parasitic plant family Orobanchaceae includes 270 species in 20 genera of root holoparasites of which the two Orobanchaceae genera, *Orobanche* and *Phelipanche* (common name broomrape), include more than 100 species, a few of which are major agricultural weeds in regions with Mediterranean climates. *Phelipanche aegyptiaca* is a major obstacle to the production of many broadleaf crops, most of them belonging to the Solanaceae, Fabaceae, Cruciferae and Umbelliferae plant families [5,6]. Outbreaks of *P. aegyptiaca* infestations occur frequently outside of the Mediterranean region and have been reported in Africa, Asia, Europe and recently in North America [7,8].

Broomrape species are extremely difficult to control during the cultivation of agricultural crops due to the intimate hidden underground association of the parasite haustoria with the roots of the host plant. The parasite acts as a strong metabolic sink that sucks assimilates, water and minerals from the host plant’s vascular system. Numerous methods have been attempted for field broomrape control including hand weeding, chemical herbicides, bioherbicides and biological agents, and naturally and genetically engineered crop resistance [5,6,9,10,11], most of them resulting in limited success under field-application conditions. The use of chemical control as reported in this study requires a highly effective and selective herbicide to control the parasite without harming the crop.

Imazapic belongs to the imidazolinone herbicide group, a subgroup of the acetolactate synthase enzyme inhibiting herbicides (ALS), systemic herbicides that hinder the production of branched-chain aliphatic amino acids necessary for protein synthesis and cell growth [12]. Some of these herbicides have been shown to kill broomrape directly in the soil and systemically via the host plant as the parasite acts as a strong sink readily absorbing the herbicides from the host plant [13,14,15]. Imazapic is a weak acid (pKa ≅ 3.6) that behaves as an anion in most soils [16]. The herbicide is highly soluble (2200 mg/L @25 °C) and its reported half-life in the soil varies from 31 to 233 days, depending upon soil characteristics and environmental conditions [17]. Imazapic is weakly adsorbed in high pH soil, while adsorption increases as pH decreases and as clay and organic matter content increase [17]. The herbicide is not volatile and there is little lateral movement of the herbicide in soil. The half-life of the herbicide on soils due to photolysis is 120 days but in aqueous solutions, imazapic is rapidly broken down by photolysis with a half-life of just one or two days [12,17,18].

The most common irrigation system in Israeli tomato production is surface located drip irrigation in which water and nutrients are directly and accurately applied to the plant root system. However, the distribution of herbicides in the soil under drip irrigation will vary under different soil conditions and irrigation regimes. The hypothesis proposed by us is that the parasitic plant *P. aegyptiaca* can be controlled efficiently and selectively via chemigation of imazapic through drip irrigation (herbigation) which enables elegant and precise delivery of the herbicide to the parasite.

The aim of this study was to test, under laboratory and greenhouse conditions, the factors involved in the behavior of imazapic in soil, and the consequential phytotoxicity of the herbicide to *P. aegyptiaca* and tomato plants, enabling the devising and refinement of a management tool that will effectively and selectively control *P. aegyptiaca* in processing tomato under field conditions.

## 2. Materials and Methods

### 2.1. Phelipanche Aegyptiaca Seed

The source of *P. aegyptiaca* seeds used in the laboratory and greenhouse studies was from a 2002 heavily infested chickpea (*Cicer arietinum*) field in Gesher Haziv, located in the Western Galilee of Israel (N33°04′07″, E35°11′00″No). Capsules were removed from mature dry broomrape inflorescences, air-dried, threshed, cleaned and stored in a plastic container at 4 °C until use.

For Petri dish and laboratory studies, seeds were disinfected by soaking in 70% ethanol for one minute and then in sodium hypochlorite 1% + ‘Tween 20′ 0.01% (by volume) for ten minutes. The seeds were then washed five times with sterilized water.

### 2.2. Imazapic Herbicide

The commercial imazapic product ‘Cadre’ 240 g/L produced by BASF Germany was used in all experiments. This herbicide is a selective systemic ALS inhibitor belonging to the imidazolinone group, registered and sold in Israel by Luxembourg Industries Ltd., Tel-Aviv 6812509, Israel.

### 2.3. LC-MS/MS Imazapic Analysis

LC-MS/MS chemical analysis of imazapic in soil and tomato roots was conducted according to protocols developed by us during this study. Imazapic analysis was carried out on a Sciex AB 3200 Qtrap LCMS using ESI. Ten μL were injected onto a Kinetex^®^ 5 μm C18 100 Å, 150 × 4.60 mm Phenomenex column. The mobile phase consisted of 30% eluent A and 70% eluent B: eluent A-0.2% acetic acid in water and eluent B- a 1:1 mixture of methanol:acetonitrile. The flow rate was 0.8 mL/min. Imazapic was identified by MRM with the following transitions: 276.1 → 231.1 and 276.1 → 86. The described analysis enables the detection of imazapic concentrations as low as 1 ppb.

### 2.4. Soil Samples Analysis

Imazapic was extracted from soil according to the following procedure: Ten ml of a water:methanol solution (70:30) were added to 3–5 g of soil. The sample was vortexed for 30 s and then shaken overnight on a reciprocal shaker. The samples were then centrifuged at an RCF of 2500× *g* and about 2 mL of the clear supernatant were transferred to an LCMS vial after filtration through a 45 μ spiral filter.

### 2.5. Tomato Root Samples Analysis

Tomato root samples were prepared for LC-MS/MS analysis in the following manner: plant material was dried in a lyophilizer and then ground with a mortar and pestle. A 0.1 g sample was taken and extracted with 5 mL of a 1:1 water:acetonitrile mixture and 2 mL of hexane. The sample was vortexed for 30 sec and then shaken overnight on a reciprocal shaker. After centrifugation, about 2 mL of the aqueous phase were transferred to an LCMS vial after filtration through a 45 μ spiral filter. LC-MS/MS analysis was carried out as described above.

### 2.6. Dose-Response of P. aegyptiaca Seed and Seedlings to Imazapic in Petri Dish

To test the susceptibility of *P. aegyptiaca* to imazapic in the very early parasite developmental stage we conducted dose-response experiments in which we applied imazapic at increasing concentrations to *P. aegyptiaca* seeds and seedlings.

The procedure for the seed germination experiment was as follows: following seed disinfection, ~100 *P. aegyptiaca* seeds were sprinkled on five cm diameter glass-microfibre filter discs, placed in Petri dishes and moistened with 700 µL sterilized water. The Petri dishes were then sealed with Parafilm, covered with aluminum foil, and placed in a 25 °C growth chamber for a pre-conditioning period. After seven days the Petri dishes were opened and treated with 0–5000 ppb imazapic solutions containing 500 µL of the synthetic strigol analog germination stimulant GR24. The Petri dishes were then re-sealed with parafilm, wrapped with aluminum foil, and placed back in the growth chamber. After an additional 14 days, *P. aegyptiaca* seed germination in each Petri dish was determined under a stereoscopic microscope: seeds in which the hypocotyl was longer than the seed length were considered germinating seeds.

The procedure for the seedling control experiment was as follows: seeds were sprinkled and treated as in the seed germination experiment but after one week only the synthetic germination stimulant GR24 was added (no imazapic). One week later, following seed germination, Petri dishes were opened and imazapic was added to the Petri dishes at the same concentrations as in the seed germination experiment. The Petri dishes were then resealed, covered and placed in the growth chamber. One week later seedling vitality was determined under the stereoscopic microscope.

All treatments and controls were replicated 5 times. Treatments were accompanied by two controls: 1. The synthetic stimulant GR24 was added to the Petri dish with no imazapic to test the potential germination of the seeds. 2. Five hundred µL of sterilized water only was added after preconditioning to test for spontaneous germination. All procedures were conducted under sterile conditions in a laminar flow hood to prevent contamination of seeds and seedlings.

### 2.7. Effect of Imazapic on P. aegyptiaca Parasitizing Tomato Roots in Polyethylene Bag Studies

**Figure 1:**
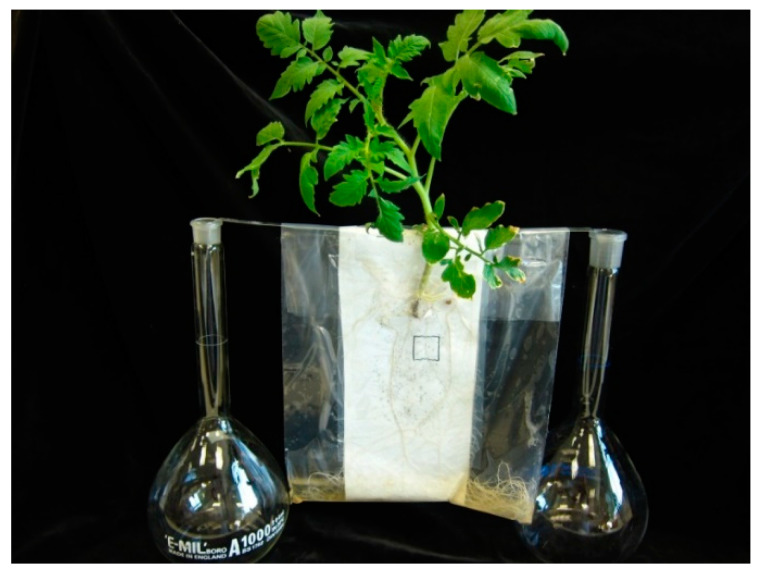
The PE bag system for studying the effect of imazapic on *P. aegyptiaca* parasitism on tomato roots shown on the day of imazapic application 36 DAP.

In addition to the Petri dish experiments in which we tested the effect of imazapic on *P. aegyptiaca* seeds, imazapic was also applied to *P. aegyptiaca* attachments and tubercles after their attachment to tomato roots in a polyethylene bag system (Goldwasser et al., 1997). This system allows us to non-destructively monitor the development of the parasite on host plant roots and the effect of the herbicide on both the parasite and the host plant. After seed disinfection (as described above), 10 mg of *P. aegyptiaca* seeds were sprinkled onto 14 by 12 cm glass-microfibre sheets (GFA paper), previously moistened with 5 mL sterile water. One 4-week-old tomato seedling was mounted on the top of each GFA sheet. Sheets were then inserted into a clear polyethylene bag (25 by 18 cm), and 100 mL of sterilized Hoagland nutrient solution was added to each PE bag (Figure 1). Polyethylene bags were hung upright in a black box so that plant roots were in the dark and their shoots projected into the air and light above the box. The box was placed in a 25 °C growth chamber and the nutrient solution was replenished in the bags as needed. Thirty-six days after planting (DAP), PE bags were emptied of the nutrient solution and imazapic was injected by a syringe to each bag mixed in 20 mL Hoagland nutrient solution at 2.5, 5 and 10 ppb, with 10 replications per treatment. The PE bags were placed in the 25 °C growth chamber and weekly monitored and recorded for broomrape and host root and foliage development.

### 2.8. Imazapic Sorption in Four Soils

To elucidate the differential efficacy of imazapic for *P. aegyptiaca* control in different soils, sorption of imazapic was studied in four soils taken from the following field sites: Ein Harod (Jezreel Valley), Eden Farm (Bet Shean), Gadash Farm (Upper Galilee) and Bet Dagan (Central Israel). Properties of the soils are presented in Table 1. Sorption was determined by the batch method: Ten ml of imazapic solution was added to 5 g of soil and shaken for 18 h on a reciprocal shaker. After centrifugation, a portion of the clear supernatant was analyzed by LC-MS/MS as described above. The initial concentrations of imazapic were 0 to 400 μg/L for the Eden Farm and Gadash Farm soils and 0 to 700 μg/L for the Ein Harod and Hamra soils. The experiment was run in duplicate and sorption was calculated based on the change in imazapic concentration in the supernatant.

### 2.9. Imazapic Dose-Response of Tomato and P. aegyptiaca in Pots

Following the imazapic dose-response of *P. aegyptiaca* seeds and seedlings in the Petri dish experiments and after attachment to tomato roots in the PE bag system, we tested the imazapic dose-response of the parasite and the host in pots in the greenhouse. The experiments were conducted in 3 L pots (19 cm diameter) filled with soil taken from the Upper Galilee Gadash Experimental Farm. *P. aegyptiaca* seeds were mixed in the soil with a cement mixer at a rate of 10 mg seeds L^−1^ soil. In each pot we planted a one-month-old tomato var. M-82 plug seedling. Imazapic was single and double drench-applied in 100 mL of water at different rates and timing of application, described in Table 2. Each treatment was replicated five times: five pots with one tomato plant each. Weekly assessment of tomato plants and broomrape inflorescences counts were performed and recorded. The experiment was terminated 71 days after tomato planting following a final count of broomrape inflorescences in each pot, cutting of each tomato plant at soil level and weighing fruit yield and foliage fresh weight for each plant.

### 2.10. Tracking Imazapic Concentration in Soil and Tomato Roots

The experiment was conducted in pots in the greenhouse as described for the dose-response experiment. Imazapic was applied 32 days after planting at 10 ppb as a single application in 100 mL water. Soil and root samples were collected from each pot 1, 3 and 7 DAA of imazapic. Soil samples and tomato root samples were prepared and analyzed for imazapic presence by LC-MS/MS as previously described.

### 2.11. Statistical Analysis

The data of each experiment was analyzed using JMP Pro^®^ statistical software, Version 14, SAS Institute Inc., Cary, NC, USA 1989–2019.

Comparison of the means of each treatment in all tables and graphs was conducted using the standard error of the mean of each treatment or by statistical analysis using the Tukey Kramer HSD test, α = 0.05 or the Student’s t LSMeans Differences test, α = 0.05.

The sorption isotherms for imazapic in different soils were determined by calculating the Kd value (L/kg), which describes the equilibrium between the herbicide concentration in the soil (mg/kg) relative to the water concentration (mg/L).

Statistical analysis of the Kd values of the different soils was determined by the Tukey Kramer HSD test, *p* = 0.01.

## 3. Results

### 3.1. Dose-Response of P. aegyptiaca Seed and Seedlings to Imazapic in Petri Dish

*P. aegyptiaca* seed germination was not affected by imazapic even at the extremely high concentration of 5000 ppb. At this herbicide dose, the parasite seeds germinated at a rate of 97–98% of the non-imazapic treated control. One-week-old *P. aegyptiaca* seedlings (with no association to the roots of a host plant) were not affected by any of the imazapic treatments as well (Table 3).

### 3.2. Effect of Imazapic on P. aegyptiaca Parasitizing Tomato Roots in Polyethylene Bag Studies

**Figure 2:**
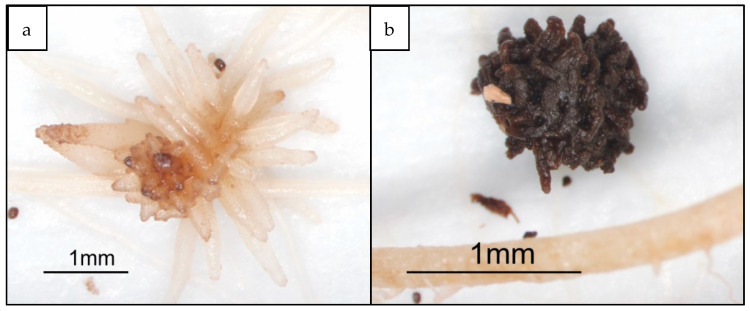
The effect of imazapic on *P. aegyptiaca* tubercles attached to tomato root in the PE bag system. (**a**)—Untreated control. (**b**)—Treated with 5 ppb imazapic.

*P. aegyptiaca* seed germination and unattached parasite seedlings were not affected by the application of imazapic, a finding in agreement with the Petri dish studies. However, once the parasite was attached to the tomato host root it became susceptible to the herbicide application at both 2.5 and 5 ppb concentrations, as determined by the senescence of the tubercle and its subsequent detachment from the tomato host root (Figure 2).

### 3.3. Imazapic Sorption to Soils

**Figure 3:**
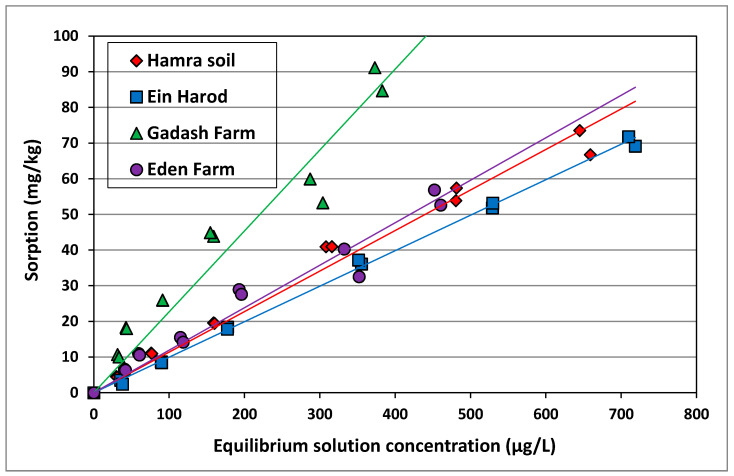
Imazapic sorption in three soils in which field studies were performed and in Hamra soil as a reference soil. All isotherms were linear.

The sorption isotherms for imazapic in the studied soils are presented in Figure 3. All isotherms were linear. The sorption coefficients Kd and the OC normalized sorption coefficients (Koc) for imazapic are presented in Table 4.

The significantly higher Kd found for the Gadash Farm soil indicates that more herbicide will be adsorbed by this soil, resulting in lower efficacy of *P. aegyptiaca* control compared to the other tested soils, possibly requiring a higher dose of imazapic for efficient parasite control. The high Kd of the Gadash farm soil can be explained by its high clay and organic matter contents. The significantly lower Kd value found for the Ein Harod soil denotes that less herbicide will be adsorbed by the soil and more herbicide is available in the soil solution, thus lower imazapic doses may be needed to control the pest in this soil compared to the other tested soils.

### 3.4. Imazapic Dose-Response of Tomato and P. aegyptiaca in Pots

**Figure 4:**
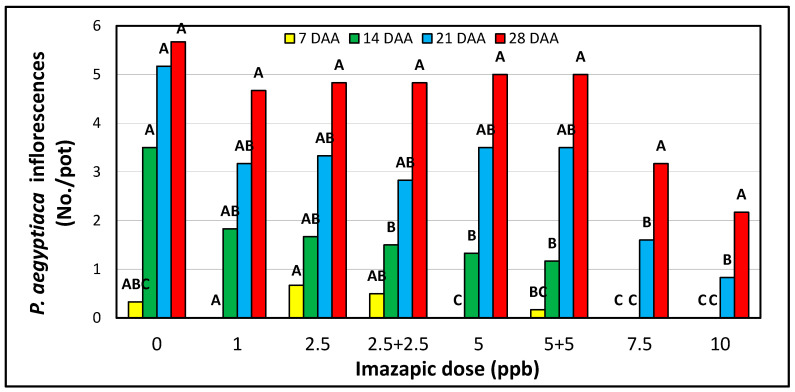
The accumulating number of emerging *P. aegyptiaca* inflorescences in the imazapic dose-response experiment in pots in the greenhouse. See Table 2 for the description of the different treatments. Letters above bars represent the statistical differences between the *P. aegyptiaca* inflorescences of each treatment analyzed by applying the Tukey-Kramer HSD test, α = 0.05. DAA-days after first imazapic application.

In the imazapic dose-response pot experiment, *P. aegyptiaca* emergence started in the 0, 2.5, 2.5 + 2.5 and 5.5 ppb treatments a few days after the first imazapic treatment (Figure 4). The number of inflorescences gradually increased in these treatments, reaching 4.7 to 5.7 inflorescences per pot 28 DAA of imazapic. In the 7.5 and 10 ppb treatments, *P. aegyptiaca* inflorescences emergence was delayed until 21 DAA and held to 1.6 and 0.83 inflorescences per pot 21 DAA, and reaching 3.17 and 2.17 at the end of the experiment, significantly lower than the infestation in the no-herbicide control treatment.

**Figure 5:**
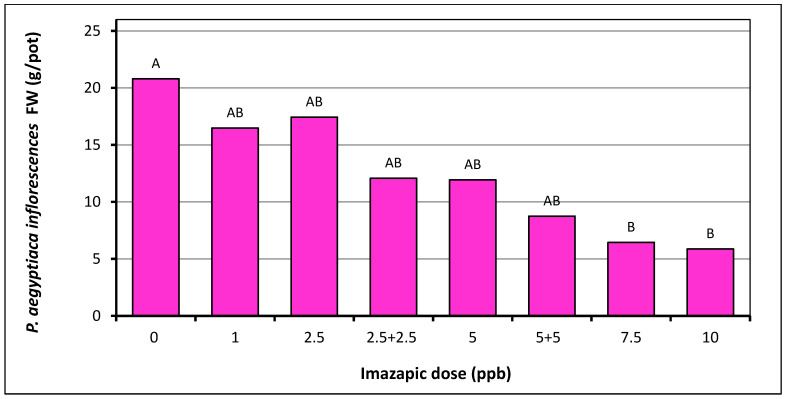
*P. aegyptiaca* aboveground fresh weight (FW) at the end of the imazapic dose-response experiment in pots in the greenhouse, as recorded 71 days after tomato planting. See Table 2 for the description of the different treatments. Letters above bars represent the statistical differences between the *P. aegyptiaca* inflorescences of each treatment analyzed by applying the Student’s t LSMeans Differences, α = 0.05.

The effect of the higher doses of imazapic was more apparent on *P. aegyptiaca* growth than on the number of parasite inflorescences, as observed by the final inflorescences fresh weight (Figure 5). The inflorescences FW decreased from 21 g per pot in the non-treated control to 16.5–17.4 g per pot in the 1 and 2.5 ppb treatments, 12 to 11.9 g in the 2.5 + 2.5 and 5 ppb treatments, and 6.5 to 5.8 g in the 7.5 and 10 ppb treatments. All treatments reduced final *P. aegyptiaca* fresh weight compared to the non-treated control, but only the higher doses of 7.5 and 10 ppb caused a statistically significant reduction.

Though phytotoxic symptoms on tomato plants were observed for imazapic concentrations above 5 ppb, fruit yield was not statistically different between all treatments (data not presented).

### 3.5. Tracking Imazapic Concentration in Soil and the Tomato Plant

The soil and root imazapic analysis indicated that 1 DAA of imazapic the herbicide accumulated in the tomato roots, reaching concentrations of 263.6 ppb. Three DAA the accumulation in the roots continued, reaching a concentration 1.7-fold higher than at 1 DAA, while in the soil it dropped 2.9 fold. On day 7 the soil concentration diminished to 0.4 ppb and the root concentration dropped but remained high at 234 ppb (Table 5).

## 4. Discussion and Conclusions

The laboratory and greenhouse experiments demonstrate the complexity of chemical control of broomrape via soil drench/chemigation with imazapic. Successful control depends on many factors, and the interactions among them: herbicide, soil, host plant, parasitic-plant, temperature and moisture. In the presented studies, some of these factors were examined under controlled conditions, enabling the transfer of efficient and selective *P. aegyptiaca* control strategies under field conditions.

In the Petri dish experiments, we elucidated that broomrape seeds and seedlings that are not attached to host plant roots are not sensitive to imazapic even at extreme concentrations. The observation in the PE bags supported this finding by showing that *P. aegyptiaca* is injured by imazapic only after attachment to the tomato host root at imazapic concentrations of 2.5 and 5 ppb in the culture solution.

Imazapic is an ionizable herbicide; thus, it can exist in the soil in two different forms depending on the pH. When the pH is below 3.6, the molecular form predominates, and as the pH increases the anion prevails and the attractive forces between the herbicide and the soil components decrease. The result is that imazapic exhibits extremely low sorption behavior in most agricultural soils [16]. The Koc values for imazapic in the four soils studied are fairly uniform. The fact that imazapic is present mainly in the ionized form in these soils due to their high pH values should rule out hydrophobic sorption by the soil organic matter (SOM) as the main mechanism of sorption. Sorption of the anionic form of imazapic may occur on the broken edges of clay minerals or on sesqui-oxides in the soils. In any case, the extremely low sorption values (Kd) are what determine the transport of imazapic in soils and they indicate that imazapic applied via drip irrigation will be highly mobile in the soil. Thus, when applied at a constant concentration the entire wetted volume of the soil will contain imazapic with the exception of a small volume at the edges of the wetted front. Subsequent irrigation without imazapic will leach the chemical to the boundaries of the wetted zone leaving an imazapic-free volume around the emitter. Similarly, if imazapic is applied as a pulse at the beginning of the irrigation cycle, it will be leached out the volume around the emitter. Similar behavior was observed for bromacil, a slightly adsorbed herbicide, in various soils [19,20]. The differences in the Kd values of imazapic in different soils as elucidated in this study can explain differences in the efficacy of the herbicide in control of *P. aegyptiaca* and has to be taken into account when devising a soil-applied herbicide-based control scheme.

In the greenhouse experiments, we elucidated that imazapic can selectively control *P. aegyptiaca* attached to tomato plant roots, but effective control requires frequent repeated applications to maintain this concentration in the soil throughout the tomato plant growth period.

The tracking of imazapic in soil and tomato roots showed that imazapic concentration in the soil rapidly diminishes, such that 7 DAA very little herbicide remains in the soil, thus requiring repeated herbicide applications. The good tolerance of the tomato host and the high phytotoxicity of imazapic to the attached parasite are due to a source-sink relationship in which the parasite acts as a strong sink absorbing most of the herbicide and thus relieving the tomato plant from phytotoxic effects. The imazapic dissipation mechanism in the soil is most probably due to microbial metabolism [17].

Numerous control methods have been attempted to effectively and selectively control broomrape under field conditions, but most of these attempts have resulted in limited or partial control. The presented laboratory and greenhouse studies have provided invaluable knowledge for devising field imazapic application strategies via soil drench or drip irrigation systems for efficient and selective broomrape control and supported the successful development of field experiments and Decision Support Systems as described by Eizenberg et al. [14], Ephrath et al. [21] and Eizenberg and Goldwasser [13].

## Figures and Tables

**Table 1:** Properties of the soils in the imazapic sorption studies.

Soil Source	pH	Clay (%)	Silt (%)	Sand (%)	OC (%)
Eden Farm	7.4	47	27	26	1.2
Gadash Farm	7.5	59	31	10	0.94
Ein Harod	8.0	57	29	14	0.41
Bet Dagan	7.6	10	3	87	0.40

OC—Soil organic carbon.

**Table 2:** Imazapic treatments in the imazapic dose-response pot experiment. DAP = days after planting.

Treatment	Imazapic Application (ppb)	
	27 DAP	51 DAP
Control		
1	1.0	
2	2.5	
3	2.5	2.5
4	5.0	
5	5.0	5.0
6	7.5	
7	10.0	

**Table 3:** Dose-response of *P. aegyptiaca* seed germination and seedling vigor to imazapic. Seed germination in the no-herbicide control treatment in which only the GR24 stimulant was added was 79.4%. No spontaneous seed germination was recorded in the no-GR24 no-imazapic control treatment. ± = Standard error.

Imazapic Concentration (ppb)	Seed Germination (% of Control)	Seedling Vigor (% of Control)
0	100.0 ± 0.01	100.0 ± 0.02
500	97.90 ± 0.04	94.98 ± 0.02
1000	96.54 ± 0.02	96.45 ± 0.03
5000	97.28 ± 0.01	98.45 ± 0.04

**Table 4:** The calculated sorption coefficients. Kd, and the organic carbon (OC) normalized sorption coefficients (Koc) for imazapic in the four tested soils. Letters following the Kd values represent statistical differences according to the Tukey HSD test, *p* = 0.01.

Soil	Kd (L/kg)	Koc	r^2^
Gadash Farm	0.23 a	24.4	0.938
Eden Farm	0.13 b	10.8	0.957
Hamra	0.11 bc	27.5	0.983
Ein Harod	0.10 c	26.8	0.998

**Table 5:** Imazapic accumulation in the soil and tomato roots in the pot experiment. The data is averaged over four replications per treatment and ± represents the standard error of the means of each treatment.

Days after Treatment	Imazapic Soil Concentration (ppb w/w)	Imazapic Tomato Root Concentration (ppb w/w)
1	7.5 ± 2.4	263.6 ± 30.5
3	2.6 ± 0.1	344.3 ± 93.9
7	0.4 ± 0.2	234.6 ± 87.3

## Data Availability

The data is contained within the article.

## References

[1] Heide-Jorgensen H.S., Joel D.M., Gressel J., Musselman L.J. (2013). Introduction: The parasitic syndrome in higher plants. Parasitic Orobanchaceae.

[2] Goldwasser Y., Rodenberg J., Joel D.M., Gressel J., Musselman L.J. (2013). Integrated Agronomic Management of Parasitic Weed Seed Banks. Parasitic Orobanchaceae.

[3] Parker C., Joel D., Gressel J., Musselman L. (2013). The Parasitic Weeds of the Orobanchaceae. Parasitic Orobanchaceae.

[4] Parker C., Riches C. (1993). Parasitic Weeds of the World: Biology and Control.

[5] Goldwasser Y., Kleifeld Y., Inderjit (2004). Recent approaches to *Orobanche* management—A review. Weed Biology and Management.

[6] Joel D.M., Gressel J., Musselman L.J. (2013). Parasitic Orobanchaceae.

[7] CABI Invasive Species Compendium-Orobanche aegyptiaca. https://www.cabi.org/isc/datasheet/37742#toidentity.

[8] Miyao G. Egyptian broomrape eradication effort in California: A progress report on the joint effort of regulators, university, tomato growers and processors. Proceedings of the XIV International Symposium on Processing Tomato, ISHS Acta Horticulturae 1159.

[9] Aparicio M.F., Delavault P., Timko M.P. (2021). Management of infection by parasitic weeds: A review. Plants.

[10] Bai J., Wei Q., Shu J., Gan Z., Li B., Yan D., Huang Z., Guo Y., Wang X., Zhang L. (2020). Exploration of resistance to *Phelipanche aegyptiaca* in tomato. Pest Manag. Sci..

[11] Goldwasser Y., Kleifeld Y., Plakhine D., Rubin B. (1997). Variation in vetch (*Vicia* spp.) response to *Orobanche aegyptiaca*. Weed Sci..

[12] Shaner D.L. (2014). Herbicide Handbook.

[13] Eizenberg H., Goldwasser Y. (2018). Control of Egyptian Broomrape in Processing Tomato: A Summary of 20 Years of Research and Successful Implementation. Plant Dis..

[14] Eizenberg H., Aly R., Cohen Y. (2012). Technologies for smart chemical control of broomrape (*Orobanche* spp. and *Phelipanche* spp.). Weed Sci..

[15] Hershenhorn Y., Eizenberg H., Dor E., Kapulnik Y., Goldwasser Y. (2009). *Phelipanche aegyptiaca* management in tomato. Weed Res..

[16] Lewis K.A., Tzilivakis J., Warner D., Green A. (2016). An international database for pesticide risk assessments and management. Hum. Ecol. Risk Assess. Int. J..

[17] Tu M., Hurd C., Randall J.M. (2001). Weed Control Methods Handbook.

[18] American Cyanamid Company (2000). Plateau Herbicide, for Weed Control, Native Grass Establishment and Turf Growth Suppression on Roadsides and Other Non-Crop Areas, PE-47015.

[19] Gerstl Z., Yaron B. (1983). Behavior of bromacil and napropamide in soils. II. Distribution after application from a point source. Soil Sci. Soc. Am. J..

[20] Gerstl Z., Albasel N. (1984). Field distribution of pesticides applied via a drip irrigation system. Irrig. Sci..

[21] Ephrath J.E., Hershenhorn J., Achdari G., Bringer S., Eizenberg H. (2012). Use of logistic equation for detection of the initial parasitism phase of Egyptian broomrape (*Phelipanche aegyptiaca*) in tomato. Weed Sci..

